# Influenza Excess Mortality from 1950–2000 in Tropical Singapore

**DOI:** 10.1371/journal.pone.0008096

**Published:** 2009-12-01

**Authors:** Vernon J. Lee, Jonathan Yap, Jimmy B. S. Ong, Kwai-Peng Chan, Raymond T. P. Lin, Siew Pang Chan, Kee Tai Goh, Yee-Sin Leo, Mark I-Cheng Chen

**Affiliations:** 1 Biodefence Center, Ministry of Defence, Singapore, Singapore; 2 Department of Clinical Epidemiology, Tan Tock Seng Hospital, Singapore, Singapore; 3 Yong Loo Lin School of Medicine, National University of Singapore, Singapore, Singapore; 4 National Center for Epidemiology and Population Health, Australian National University, Canberra, Australia; 5 Department of Pathology, Singapore General Hospital, Singapore, Singapore; 6 Ministry of Health, Singapore, Singapore; 7 SIM University, Singapore, Singapore; 8 Department of Infectious Diseases, Tan Tock Seng Hospital, Singapore, Singapore; 9 Duke-NUS Graduate Medical School, Singapore, Singapore; Singapore Immunology Network, Singapore

## Abstract

**Introduction:**

Tropical regions have been shown to exhibit different influenza seasonal patterns compared to their temperate counterparts. However, there is little information about the burden of annual tropical influenza epidemics across time, and the relationship between tropical influenza epidemics compared with other regions.

**Methods:**

Data on monthly national mortality and population was obtained from 1947 to 2003 in Singapore. To determine excess mortality for each month, we used a moving average analysis for each month from 1950 to 2000. From 1972, influenza viral surveillance data was available. Before 1972, information was obtained from serial annual government reports, peer-reviewed journal articles and press articles.

**Results:**

The influenza pandemics of 1957 and 1968 resulted in substantial mortality. In addition, there were 20 other time points with significant excess mortality. Of the 12 periods with significant excess mortality post-1972, only one point (1988) did not correspond to a recorded influenza activity. For the 8 periods with significant excess mortality periods before 1972 excluding the pandemic years, 2 years (1951 and 1953) had newspaper reports of increased pneumonia deaths. Excess mortality could be observed in almost all periods with recorded influenza outbreaks but did not always exceed the 95% confidence limits of the baseline mortality rate.

**Conclusion:**

Influenza epidemics were the likely cause of most excess mortality periods in post-war tropical Singapore, although not every epidemic resulted in high mortality. It is therefore important to have good influenza surveillance systems in place to detect influenza activity.

## Introduction

Tropical regions have been shown to exhibit different influenza seasonal patterns compared to temperate regions. While temperate countries have a single annual epidemic during winter, influenza in the tropics spreads throughout the year, with two annual peaks having been described for Singapore, a globally-connected tropical city [1], [2]. However, while the seasonality differs from temperate countries, mortality from influenza activity in tropical Singapore is comparable to temperate and sub-tropical countries such as the United States and Hong Kong [3]. The three 20^th^ century influenza pandemics in Singapore were also associated with substantial excess deaths when compared against baseline mortality rates in surrounding years [4].

Long time-series data on successive influenza seasons have been used to highlight and quantify the burden of disease attributable to influenza in temperate countries [5]–[8]. In addition, such data has been used to grade the severity of different epidemic influenza seasons as well as specific influenza sub-types and strains [9]–[11]. However, there are few equivalent studies on the burden of annual influenza epidemics in the tropics, and the relationship between tropical influenza epidemics compared with other regions [12]. There is a scarcity of data on tropical influenza, due to the lack of clear seasonality and virological data to identify periods of influenza activity and its associated impact on mortality.

Singapore is a tropical island city-state in South-East Asia. Being a globally-connected city, it provides a representation of the spread of influenza in the tropics. In addition, Singapore has good consistent records of mortality statistics, and had been routinely isolating influenza viruses for surveillance since 1972 (as a nationwide study in 1972 and 1973, and as a national surveillance programme from 1974 onwards) [1]. In this study, we explore the possible links between excess mortality from 1950 to 2000 in the post-World War Two era in Singapore and influenza epidemics. This time-period also included almost 30 years of influenza virological surveillance data. We use this data to demonstrate the clear correlation between influenza epidemic periods and excess mortality, and highlight the burden and timing of prominent influenza epidemics in tropical Singapore.

## Materials and Methods

Data on monthly national all cause mortality and population size was obtained from 1947 to 2003 from the Registry of Births and Deaths, Singapore through the Department of Statistics, Singapore–the governmental agency responsible for collection, verification, and maintenance of national statistics. Monthly mortality rates were calculated from this data.

To determine the excess mortality for each month from 1950 to 2000, we used a moving average analysis which has proven appropriateness due to the lack of distinct seasonal mortality patterns in Singapore [4]. Unlike temperate regions, where methods relying on seasonal variation such as that used by Serfling are commonly used, we assumed that monthly mortality in Singapore exhibited a secular trend without seasonal components. We therefore used a moving average analysis for each month constructed using data from 3 years before and 3 years after the month (excluding the month itself) to calculate the predicted mean and 95% confidence intervals for the expected mortality for that month. Months previously known to be affected by the 1957 and 1968 pandemics (May 1957, and August and September 1968) were excluded to eliminate inflation of confidence intervals in the periods surrounding those months. The moving averages formed the entire baseline mortality rate with 95% confidence intervals across 50 years from 1950 to 2000. Excess deaths were calculated as the actual mortality rate on record minus the moving average baseline rate. Months for which the mortality rate exceeded the 95% confidence intervals were considered as those with significant excess deaths, and used to highlight possible influenza epidemic periods. The analyses were performed in Stata 10.0 for Windows (Stata Corp., College Station, TX, USA).

Data on influenza virological surveillance was obtained from the Department of Pathology at the Singapore General Hospital, which is the World Health Organization (WHO) National Influenza Centre (NIC) in Singapore since 1972 [13]. The surveillance programme tracks influenza activity year-round through virus isolation and identification from respiratory samples collected from patients attending government outpatient clinics for influenza-like symptoms, as well as from patients in hospitals and private clinics. Strain characterization was performed at the WHO Collaborating Centres for Reference and Research on Influenza in the USA, UK and Australia, and at the NIC. The data included records of predominant strains and their periods of circulation in Singapore, which were available from 1972 onwards. The proportion of respiratory illness samples positive for influenza was available from 1972 to 1993, and the breakdown on the percentage of samples positive for influenza A (H1N1), (H3N2), and influenza B was available from 1994 onwards. These proportions were compared to mortality rates to determine if periods with excess mortality corresponded to increases in influenza isolates.

In addition, we performed a search of serial annual government reports from the Department of Health, Singapore from 1950 to 1965 (before Singapore gained independence), the Ministry of Health and the Ministry of the Environment from 1965 onwards (post-independence), peer-reviewed journal articles, and press articles from The Straits Times (the main and only English newspaper across all the years). This was done for all the months where significant excess mortality occurred, to determine if there were reports suggestive of influenza epidemics. This provided additional evidence of the known influenza outbreaks, and the recorded burden of these epidemics.

## Results

**Figure 1:**
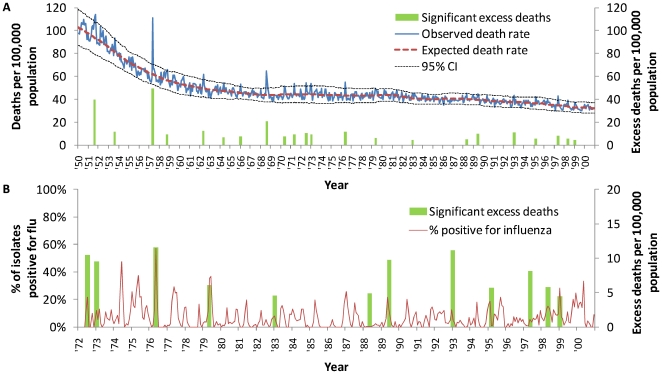
Periods of significant excess deaths in tropical Singapore*. Panel A–Excess deaths compared to overall deaths, 1950 to 2000. Panel B–Excess deaths compared to positive influenza positive, 1972 to 2000. *Significant excess mortality which occurred over 2 contiguous months (August to September 1951, June to July 1989, and December 1992 to January 1993) was summed to allow for comparisons of overall epidemic magnitude.

The annual number of deaths from 1950 to 2000, and excess deaths where mortality exceeded the upper limit of the 95% confidence interval is shown in Figure 1A. Apart from the known pandemics of 1957 and 1968, which resulted in substantial mortality, there were 20 other time points in which there was significant excess mortality.

Excluding the two pandemics, there were 8 periods with excess mortality before 1972. Of these, 2 periods coincided with newspaper reports of increased pneumonia deaths. There were 3 weeks in August 1953 where pneumonia was the mentioned as the main cause of deaths in newspaper reports (weeks ending August 8 with 23 deaths, August 15 with 30 deaths, and August 22 with 31 deaths) [14], [15]. In August and September 1951, pneumonia was also the main cause of deaths for the weeks ending August 18 (29 deaths), August 25 (30 deaths), September 2 (35 deaths) [16]–[18]. In end-September 1951, there was also an increase in pneumonia cases in Malaysia (a country North of Singapore also under the administration of the British Empire at the time) [19].

**Figure 2:**
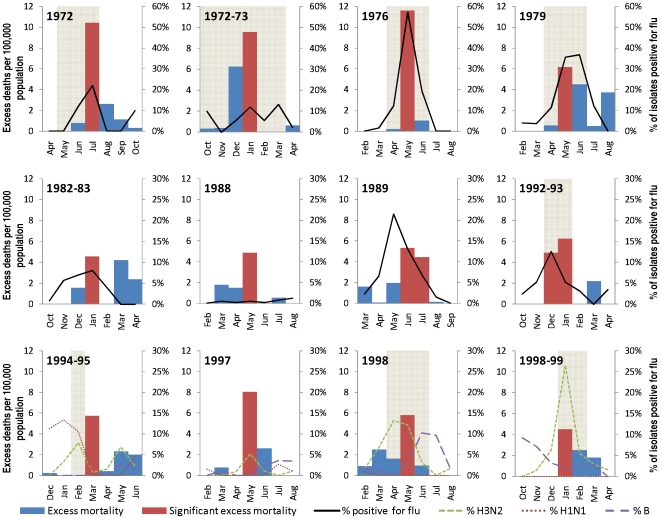
Periods with significant excess deaths in Singapore and positive influenza isolates, 1972 to 2000*. *Areas shaded in grey correspond to official reports of influenza epidemics during the time period.

Figure 1B shows the significant excess mortality periods and the % of respiratory illness sample isolates positive for influenza from 1972 onwards when virological data became available. There were 12 periods with significant excess mortality post 1972 for which virological data was available. Figure 2 shows the excess mortality and corresponding influenza virological surveillance for these periods. Of the 12 periods with excess mortality post 1972, only one (1988) did not coincide with a temporal increase in the percentage of respiratory illness samples that were positive for influenza, although only 8 of the 12 periods were explicitly labeled as influenza epidemics or outbreaks on government records.

Table 1 lists all time periods with significant excess mortality, along with periods described as influenza epidemics or outbreaks on government records. Over the 29 years (1972 to 2000) for which influenza surveillance records are described, there were 21 influenza epidemic periods, and an additional 4 periods where significant excess mortality was observed (three of which corresponded to an increase in virological activity). Of the 12 periods of significant excess mortality post-1972, 5 of the peak excess mortality months occurred in the month of May, followed by 4 in January, and 1 each in March, June, and July. Of the 13 epidemic periods which did not result in significant excess mortality, 5 epidemic peaks occurred in the month of May, with 2 peaks each in January and July, and one each in Mar, April, June, and October. The month of May also dominated in the 8 non-pandemic significant excess mortality periods pre-1972, with 4 occurring in that month, and 1 each in January, August, September, and October. Overall, there were more reported increases in May compared to all other months.

**Table 1:** All reported influenza epidemics and months with excess mortality in Singapore, 1972 onwards.

Reported influenza epidemic period	Peak excess mortality month*	Overall excess mortality†	Excess mortality rate per 100,000†	Dominant influenza strains during epidemic period
May 72–Jul 72	Jul 72	294.7	13.8	A/England/42/72 (H3N2), 1^st^ wave‡
Oct 72–Mar 73	Jan 73	341.2	15.8	A/England/42/72 (H3N2), 2^nd^ wave‡
May 74–Jul 74	May 74	218.1	9.9	A/Port Chalmers/1/73 (H3N2), 1^st^ wave
Nov 74–Feb 75	Jan 75	3.5	0.2	A/Port Chalmers/1/73 (H3N2), 2^nd^ wave
Apr 75–Jun 75	May 75	54.9	2.4	A/Scotland/840/74 (H3N2)
Jul 75–Jul 75	Jul 75	12.4	0.6	B/Hong Kong/5/72
Apr 76–Jun 76	May 76	291.7	12.8	A/Victoria/3/75 (H3N2)‡
Apr 77–Jul 77	May 77	160.0	6.9	A/Victoria/3/75 (H3N2), A/Texas/1/77 (H3N2) and B/Hong Kong/5/72
Dec 77–Feb 78	Jan 78	82.4	3.5	A/USSR/1/77 (H1N1)
Sep 78–Oct 78	Oct 78	59.4	2.5	A/Brazil/11/78 (H1N1)
Apr 79–Jun 79	May 79	264.2	11.2	B/Singapore/222/79‡
Apr 80–Jun 80	May 80	121.4	5.1	A/Texas/1/77 (H3N2)
May 81–Jun 81	Jun 81	121.4	4.9	A/England/333/80 (H1N1)
-	Jan 83	162.4	6.1	A/Philippines/2/82 (H3N2)‡
May 83–Jul 83	May 83	93.0	3.5	A/Chile/1/83 (H1N1)
Jul 84–Sep 84	Jul 84	136.4	5.0	A/Philippines/2/82 (H3N2)
Apr 85–Jun 85	Apr 85	115.5	4.2	A/Philippines/2/82 (H3N2)
Mar 86–May 86	Mar 86	84.7	3.1	A/Switzerland/79/85 (H1N1), A/Dunedin/27/84 (H1N1), A/Victoria/7/83 (H1N1) and A/Singapore/6/86 (H1N1)
-	May 88	179.3	6.4	A/Victoria/7/87 (H3N2), A/Sichuan/2/87 (H3N2), A/Sydney1/87 (H3N2) and B/Victoria/2/87‡
-	Jun 89	339.2	11.7	A/Shanghai/11/87-like (H3N2) and A/OMS/5389/88-like (H3N2)‡
Dec 92–Jan 93	Jan 93	361.0	11.2	A/Beijing/32/92 (H3N2)‡
Feb 95–Mar 95	Mar 95	212.9	6.2	A/Taiwan/86 (H1N1), A/Texas/36/91 (H1N1)‡
-	May 97	398.0	10.7	A/Wuhan/359/95 (H3N2)‡
Apr 98–Jun 98	May 98	324.1	8.4	A/Sydney/5/97 (H3N2), 1^st^ wave‡
Jan 99–Feb 99	Jan 99	275.2	7.0	A/Sydney/5/97 (H3N2), 2^nd^ wave‡

*Month with highest excess mortality during a reported influenza epidemic period, or month with highest excess mortality in a period with significant excess mortality.

†Sum of positive deviations from the expected mortality for three-month period centered around the month with peak excess mortality; excess mortality rate is derived using estimates for total Singapore population during that period.

‡Periods with significant excess mortality.

Influenza epidemics often accompanied the introduction of new influenza antigenic variants, and new influenza variants also often gave rise to second epidemic waves. The spread of H1N1 following its re-emergence in late 1977 followed such a pattern, with the first wave causing epidemics from Dec 77 to Feb 78 (as A/USSR/1/77), followed by a second wave which passed through Singapore from Sep 78 to Oct 78 (as A/Brazil/11/78). In most cases, second waves had less mortality than the first, the exception being A/England/42/72, where both epidemic waves caused substantial excess mortality, with the first wave which peaked in Jul 72 being milder than the second wave which peaked six months later in Jan 73.

**Figure 3:**
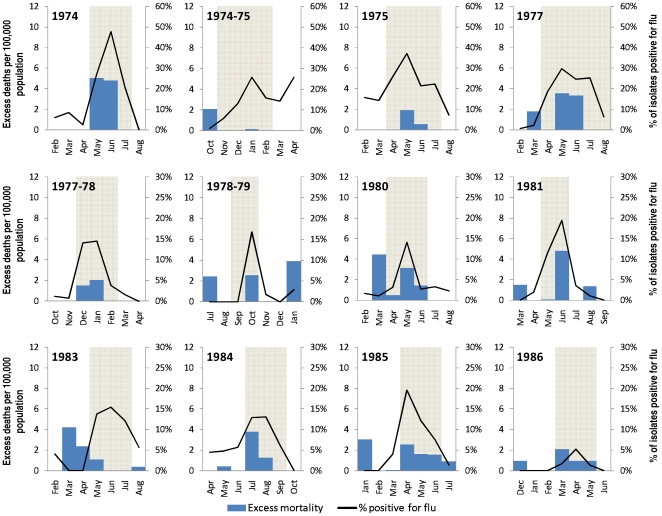
Additional epidemic periods in Singapore, with corresponding excess deaths and positive influenza isolates, 1972 to 2000*. *Areas shaded in grey correspond to official reports of influenza epidemics during the time period.

Epidemic periods which did not give rise to significant excess mortality caused lower overall excess mortality, although positive deviations from baseline mortality were detectable for most periods for which epidemic activity had been reported (Figure 3). Temporal peaks in mortality either corresponded to or lagged by one month the peaks in percentage of respiratory illness isolates positive for influenza.

Table 2 compares the epidemic timing and excess mortality attributable to prominent epidemics (the two pandemics (1957 and 1968), H1N1 emergence in 1977, and 1951 where pneumonia deaths contributing substantially to overall mortality) to the 10 time periods with most excess mortality after 1972 when virological data became available. With the exception of the A/Port Chalmers/1/73 (H3N2) epidemic in May 1974, 9 of the 10 periods also qualified as periods of significant excess mortality. Excess deaths in Aug-Sep 1951 (46 excess deaths per 100,000) were comparable to the 1957 pandemic (54 excess deaths per 100,000) while the H3N2 pandemic of 1968 caused half as much excess mortality. Estimates for the most severe influenza epidemic seasons ranged from 7.0 to 15.8 excess deaths per 100,000 population. With the exception of B/Singapore/222/79, H3N2 influenza activity featured in all the other 9 time periods. The re-emergence of H1N1 influenza as A/USSR/1/77 caused relatively mild mortality with only 3.5 excess deaths per 100,000 population; this was compared to a mean of 11.3 for the 10 episodes featured in Table 2, and 6.9 for all the periods listed in Table 1. Of the 10 periods with the most severe excess mortality, half occurred in the month of May; three others occurred in January, with one each in Jun and Jul.

**Table 2:** Ten most severe recorded influenza epidemic seasons in Singapore from 1972 to 2000 and selected countries with similar epidemics during the period, in comparison with selected influenza pandemics and epidemics.

	Excess mortality per 100,000	Month with excess peak mortality	Selected countries affected by the same virus during the same period, with timing of epidemics in parentheses. Excess all cause mortality per 100,000 are shown for selected outbreaks in the US and England & Wales where data is available
Ten most severe recorded influenza epidemic seasons			
A/England/42/72 (H3N2), 1^st^ wave	13.8	Jul 1972	India (1971–72), Nepal (1972–1973) [34] Australia (August 72) [20] England & Wales (1972–73) [21] United States (1972–73) [6], [10]–Excess mortality 9.0
A/England/42/72 (H3N2), 2^nd^ wave	15.8	Jan 1973	As above.
A/Port Chalmers/1/73 (H3N2), 1^st^ wave	9.9	May 1974	Port Chalmers, New Zealand (1973) [22] Nigeria (1974) United States (1974–75) [6], [10] - Excess mortality 7.0 Houston, United States (1974–75) [35]
A/Victoria/3/75 (H3N2)	12.8	May 1976	United States (1975–76) [6], [10] - Excess mortality 11.4 Houston, United States (1976) [36], [37] England & Wales (1975–76) [25] - Excess mortality 60.8
B/Singapore/222/79	11.2	May 1979	United States (1979–80) [6], [10] - Excess mortality 7.6 Houston, United States (1979–80) [35] England & Wales (1978–79) [25] - Excess mortality 16.3
A/Shanghai/11/87-like, A/OMS/5389/88-like (H3N2)	11.7	Jun 1989	USA (1989–90) (A/Shanghai/11/87) [38] - Excess mortality 4.2 [6], [10] Poland (1990) (A/OMS/5389/88) [39] England & Wales (1988–89) [25] - Excess mortality 20.0
A/Beijing/32/92 (H3N2)	11.2	Jan 1993	USA (1991–92) [10] - Excess mortality 16.7 Netherlands (1993–94) [40] Ontario, Canada (1993–94) [41] Lasi, Romania (1993–94) [42]
A/Wuhan/359/95 (H3N2)	10.7	May 1997	USA (1996–97) [11] - Excess mortality 25.9 Pune, India (Jan–Feb 1996) [44] Poland (Feb 1997) [45] Thailand (Jul–Aug 1997) [46]
A/Sydney/5/97 (H3N2), 1^st^ wave	8.4	May 1998	USA (1997–98) [11], [47] - Excess mortality 26.8 Australia (1997) [24] South Africa (1998) [25]
A/Sydney/5/97 (H3N2), 2^nd^ wave	7.0	Jan 1999	USA (1998–99) [11] - Excess mortality 23.1
Other epidemics and pandemics of note in Singapore			
1951 influenza epidemic strain (H1N1)	46.4	Sep 1951	
Asian influenza pandemic (H2N2)	54.4	May 1957	
Hong Kong influenza pandemic (H3N2)	28.0	Aug 1968	
A/USSR/1/77 (H1N1)	3.5	Jan 1977	

Most of the key antigenic drift variants which caused severe epidemics in Singapore also caused epidemics elsewhere (Table 2). In 1951, 1972/3, 1976, and 1998/99, the periods of high mortality corresponded to some of the highest non-pandemic mortality periods [20]. However, the relative mortality of individual epidemics vary in different countries. The 1975–76 epidemic in England and Wales due to A/Victoria/3/75 (H3N2) had an excess mortality of 60.84/100000 (37), which was much higher than similar epidemics in Singapore and the US (10). In Singapore and the US, most of the outbreaks caused by the similar viruses in similar time period had generally comparable excess mortality rates (see Table 2). However, the A/Sydney/5/97 (H3N2) epidemics in the US had a much higher mortality of 26.82 and 26.10 per 100,000 population in the winters of 1997–98 and 1998–99 respectively (11). This was higher compared to that of Singapore and as well as many previous epidemics in the US (11). The timing of occurrence in different countries also varies. The first wave of infections with A/England/42/72 (H3N2)-related viruses peaked in Singapore in July 1972; while infections peaked about the same time in Australia in August 1972, excess mortality was not markedly high [21]. In the Northern hemisphere winter of 1972 to 1973, relatively severe epidemics of A/England/42/72 (H3N2) were noted on both sides of the Atlantic Ocean [6], [22]. Singapore also experienced a severe second wave which peaked in Jan 73. For influenza A/Port Chalmers/1/73 (H3N2), outbreak reports in New Zealand dated to Sept 1973 [23] but the first wave in Singapore only peaked in May 1974, and related viruses did not cause substantial mortality in Northern Hemisphere countries like the USA until the winter of 1974 to 1975 [6]. The epidemic of B/Singapore/222/79 in May 1979 also heralded the epidemic in the USA in the winter of 1979 to 1980 [24]. Conversely, A/Victoria/3/75 (H3N2) related viruses caused a severe influenza season in the USA and England and Wales in the winter of 1975 to 1976 with excess mortality of 11.39 and 60.84 per 100,000 population respectively [6], [25] before the first wave spread through Singapore in April to June 1976. While A/Sydney/5/97 (H3N2) infections were reported in Australia in the mid-1997 influenza season, this was followed by the spread in the Northern hemisphere [11], [26] before major epidemics in Singapore and some other countries [27] in the earlier half of 1998.

## Discussion

Influenza possibly accounted for the majority of time periods with significant excess mortality in tropical Singapore in the post-war years, including the 3 highest mortality records in 1951, 1957, and 1968. Although most pre-1972 significant excess mortality months lacked documented evidence of influenza epidemics, two periods (August and Sept 1951, and August 1953) coincided with media reports of pneumonia deaths, and an additional four periods occurred in May–the month with the most number of known influenza epidemic peaks. This suggests that influenza probably resulted in more excess mortality than any other variable cause. The second highest excess mortality in 1951 was only slightly less than the 1957 pandemic and 70% higher than the 1968 pandemic. This coincided with 3 consecutive newspaper reports of pneumonia dominating mortality [16]–[18], and excess pneumonia deaths correlating with increased all-cause mortality are known to occur during influenza epidemics [10]. The year 1951 also saw major influenza outbreaks caused by the influenza A (H1N1) virus, with higher mortality and transmissibility in England and Wales and Canada than the 1957 and 1968 pandemics [28]. In Liverpool, the supposed epicenter of the 1951 epidemic, severity was higher than the 1918 pandemic [29]. Our data strongly suggests that the burden of the 1951 epidemic was not restricted to temperate regions but also affected tropical countries like Singapore and Malaysia.

In contrast, the large dengue outbreaks in the 1990s and 2000s, which where constantly in the media reports, resulted in much fewer deaths [30]–[32]; while the worst industrial accident in Singapore's history, the Spyros oil tanker explosion, killed only 76 people. The number of deaths attributable to each significant influenza epidemic was much higher than that caused by any other known man-made or natural cause during the same period. Based on this observation, it is therefore of public health and socio-economic importance to have good surveillance and prevention programs against seasonal influenza. Future measures should include promotion of annual influenza vaccination, and early epidemic identification and virological surveillance to allow judicious use of measures such as anti-viral treatment of influenza during epidemic periods. Vaccine utilization in Singapore has hitherto been poor (<0.5% of the population), making vaccine mismatch less relevant [3]. In concert with promoting seasonal vaccination programs, studies are needed to explore vaccine match to tropical epidemic strains. Other studies should also be performed to validate the utility of different surveillance systems, and determine the cost-effectiveness of surveillance and intervention programs.

While our simple algorithm for flagging months with significant excess mortality appears to have a high specificity for detecting periods of influenza activity, not all significant epidemics were identified by the algorithm. For instance, the first wave of the A/Port Chalmers/1/73(H3N2) virus which peaked in May 1974 caused substantial excess mortality (Table 2), but did not exceed the 95% confidence limits for that period, possibly because it was flanked by several epidemics of equal or greater severity (A/England/42/72 in Jul 1972 and Jan 1973, and A/Victoria/3/75 (H3N2) in May 1976). Some epidemics may also have had high morbidity with low mortality such as the A/USSR/90/77(H1N1) strains, which re-emerged in November 1977. In Singapore, infections were reported among military personnel in mid-December 1977 and spread quickly, with the epidemic peaking in January 1978 and lasting until February 1978. Government reports indicated that although outpatient attendances for upper respiratory tract infections (URTIs) doubled in January 1978, there was no corresponding increase in pneumonia and influenza (P&I) deaths [1]. As the virus affected mainly children and young adults, the relative sparing of older populations might explain the lower overall mortality of the 1977 H1N1 epidemic. Age-related immune protection in older individuals (2009) might also cause the influenza A H1N1-2009 pandemic to have a lower overall mortality rate (US CDC, 2009).

Our data also suggests the importance of correlating viral characteristics with mortality. As the 1951 epidemic suggests, epidemic seasons around that time were relatively mild in the United States in terms of morbidity and mortality, but were far more severe in Canada, England and Wales [28] and Singapore and Malaysia. In addition, in the post-1972 period, new influenza variants were noted to cause epidemics in Singapore, at times preceding (e.g. B/Singapore/222/79) and at other times following outbreaks and epidemics in other countries (e.g. A/Sydney/5/97). A systematic review comparing the burden and relative timing of different influenza seasons with a focus on identifiable strains could yield deep insights into how influenza circulates globally, with important ramifications for vaccine strain selection.

For now, the reasons for the asynchronous nature of the timing of global influenza epidemics and their differential burden of disease in different regions, as well as the driving force behind the timing of influenza epidemics in Singapore remains a mystery. Although there is little climatic seasonal variation in Singapore, this study shows that influenza seasons tend to occur from April to July and November to February, with May having most epidemics and peak excess mortality. The observed seasonality could be due to subtle differences in climate, new strains developing in tropical areas during the corresponding periods, or the spread of viruses from temperate regions. Herald waves which occur during the spring in Northern Hemisphere temperate countries [24] and taper off during the summer months, while failing to propagate in temperate countries, could go on to cause epidemics in tropical countries which are receptive to influenza viruses throughout the year.

Limitations of our study include the use of monthly all cause data which may under-estimate the burden of epidemics that straddle 2 to 3 months, as mortality split across more than 1 time period may not appear significant compared to epidemics where mortality is concentrated within the time period of analysis. The moving average calculation of the confidence intervals also means that less severe epidemics may be missed if flanked by more severe epidemics, as was the case for the 1974 epidemic caused by influenza A/Port Chalmers/1/73. Data resolution is another issue as there was no age-specific or influenza-specific data available across all 50 years. The accuracy of disease burden estimates is also difficult to assess, and different methods should be used for comparison. For example, a study in Hong Kong Island by Chiu and colleagues [33] determined influenza hospitalization rates through virological testing of hospitalized respiratory cases. Virologic data is also imperfect, as it represents only % of samples tested and not the actual size of the epidemic. As such, future studies will compare the range of estimates obtained through influenza-specific mortality, and different data sources such as primary care data and virologic testing of hospitalized or fatal cases where available. Finally, comparisons of disease burden across countries are difficult due to different methods used and future studies should analyze global data simultaneously.

Nevertheless, this study shows that crude mortality estimates can be sufficient to signal the most significant influenza epidemics, and can be easily applied to countries where data of finer resolution may be lacking. International comparisons of East Asian and tropical Southeast Asian countries should be considered, particularly at new variants in view of recent phylogenetic work which suggests that the region may be critical in the genesis of new H3N2 influenza strains [34].

Influenza epidemics were the likely cause of most of the excess mortality periods in post-war tropical Singapore, although not every influenza epidemic resulted in high mortality. It is important to have good public health programs in place to detect influenza activity. Such programmes, along with appropriate public health interventions like vaccination and judicious antiviral use, could potentially reduce the burden of seasonal or pandemic influenza.
